# Pathological mechanisms and treatment of sporadic Parkinson’s disease: past, present, and future

**DOI:** 10.1007/s00702-024-02788-w

**Published:** 2024-06-12

**Authors:** Hideki Mochizuki

**Affiliations:** Department of Neurology, Graduate School of Medicine, Osaka University, 2-2 Yamadaoka, Suita, Osaka, 565-0871 Japan

**Keywords:** Parkinson’s disease, Mitochondria, Iron, Apoptosis, α-Synuclein, Antisense oligonucleotide

## Abstract

For a special issue, we review studies on the pathogenesis of nigral cell death and the treatment of sporadic Parkinson’s disease (sPD) over the past few decades, with a focus on the studies performed by Prof. Mizuno and our group. Prof. Mizuno proposed the initial concept that mitochondrial function may be impaired in sPD. When working at Jichi Medical School, he found a decrease in complex I of the mitochondrial electron transfer complex in the substantia nigra of patients with Parkinson’s disease (PD) and MPTP models. After moving to Juntendo University as a professor and chairman, he continued to study the mechanisms of cell death in the substantia nigra of patients with sPD. Under his supervision, I studied the relationships between PD and apoptosis, PD and iron involvement, mitochondrial dysfunction and apoptosis, and PD and neuroinflammation. Moving to Kitasato University, we focused on PD and the cytotoxicity of alpha synuclein (αSyn) as well as brain neuropathology. Eventually, I moved to Osaka University, where I continued working on PD and αSyn projects to promote therapeutic research. In this paper, we present the details of these studies in the following order: past, present, and future.

## Introduction

Significant progress has been made in understanding the causes, pathogenesis, and nature of cell death in Parkinson’s disease (PD) (Figure). However, the exact mechanisms underlying sporadic PD (sPD) remain unknown. The neuropathological hallmark of PD is the loss of dopaminergic neurons predominantly in the substantia nigra pars compacta (SNpc) and the presence of intracellular inclusions termed Lewy bodies (LBs), which are mainly composed of the protein, α-synuclein (αSyn). Moreover, to investigate the mechanism of cell death in PD, Prof. Mizuno started studies on mitochondrial involvement in PD. At the time, 1-methyl-4-phenyl-l,2,3,6-tetrahydropyridine (MPTP) was discovered as a contaminant of synthetic heroin, which caused severe and irreversible parkinsonian syndrome in a number of drug abusers. The discovery of MPTP-induced parkinsonism has initiated a renaissance in basic research on PD, owing to the potential of this toxin in creating a valid disease model. Moreover, the mechanism by which MPTP causes PD is at the forefront of research.

### Mitochondria and Parkinson’s disease

MPTP-induced parkinsonism remains one of the best models of PD. Selective toxicity to the nigrostriatal dopaminergic neurons is thought to depend on the presence of an energy-dependent active uptake system of the l-methyl-4-phenyl-pyridinium ion (MPP^+^), the oxidation product of MPTP, in dopaminergic neurons. MPP^+^ is responsible for toxicity in dopaminergic neurons. Dr. Mizuno and his group were interested in the role of mitochondria as a site of action for MPP^+^ due to the structural similarity between MPP^+^ and NAD^+^, which serves as a co-factor for many respiratory enzymes. He reported, using polarographic methods, that MPP^+^ inhibits the oxidation of substrates of NAD^+^-linked dehydrogenases in the tricarboxylic acid cycle (Mizuno et al. 1987a). He also reported the inhibition of mitochondrial nicotinamide adenine dinucleotide (NADH)-ubiquinone oxidoreductase (complex I) and alpha-ketoglutarate dehydrogenase activity by MPP^+^ (Mizuno et al. 1987b). Furthermore, he showed inhibition of ATP synthesis by MPP^+^ in cerebral mitochondria incubated with glutamate + malate and ADP (Mizuno et al. 1987d). Energy crisis is considered one of the most important mechanisms of neuronal degeneration in MPTP-induced parkinsonism (Mizuno et al. 1987c, 1988a, b). Next, he examined mitochondrial function in the PD brain and demonstrated a decrease in complex I of the mitochondrial electron transfer complex by immunoblotting (Mizuno et al. 1989).

In 1989, he moved to Juntendo University as a professor and chairman of the Department of Neurology. He hypothesized that mitochondrial function might be impaired in sPD and persisted in studying mitochondrial changes in the brains of patients with PD. Dr. Hattori recapitulated immunohistochemically, the reduction of complex I in the mitochondrial electron transfer complex in the PD brain (Hattori et al. 1991). Prof. Mizuno also performed immunohistochemical studies of the mitochondrial alpha-ketoglutarate dehydrogenase complex (KGDHC) in the substantia nigra (SN) of patients with PD and showed that, in general, a decrease in KGDHC immunostaining correlated with the severity of degeneration (Mizuno et al. 1994). Taken together, the biochemical changes in PD are essentially similar to those in MPTP-induced parkinsonism.

### Iron and Parkinson’s disease

Although the initial cause of PD is not clearly defined, iron deposition has long been implicated in its etiology. There is a fairly selective and significant elevation in iron content in the SN of patients with PD, where the selective loss of dopaminergic neurons occurs. Neuromelanin (NM) readily, but not exclusively, chelates metals, particularly iron. The first research project assigned to me by Prof. Mizuno was to investigate the mechanisms of iron and melanin in the death of dopaminergic neurons. An in vitro study using a nigrostriatal co-culture demonstrated the induction of neurotoxic effects and lipid peroxidation by the iron–melanin complex in dopaminergic neurons (Mochizuki et al. 1993). Based on this data, a protective role of NM can be postulated until the buffering capacity toward iron is exhausted.

Prof. Mizuno has raised concerns about whether iron deposition is the initial cause of SN degeneration or merely a consequence. To answer this question, we used a monkey model of hemiparkinsonism generated by unilateral injection of MPTP into the caudate or putamen nuclei and compared the iron content of the SN and other basal ganglia using immunohistochemistry (Mochizuki et al. 1994a). Results showed that injection of MPTP into the caudate nucleus or putamen caused a significant increase in ferric iron reaction products in the ipsilateral SN pars compacta, indicating that injury to the nigrostriatal system after MPTP injection induced iron accumulation in the SN. In the same model, immunohistochemistry with an antibody against L-ferritin confirmed ferritin expression (Goto et al. 1996). Interestingly, immunostaining for ferritin on the SN showed no significant difference between the injected and non-injected sides; immunostaining for ferritin on the side injected with MPTP was normal, suggesting that iron accumulation in this model is unrelated to metabolic changes in L-ferritin. We have shown that dopaminergic cell death induced by MPTP administration causes secondary iron accumulation in the monkey brain, and that increased iron contributes to the progression of SN degeneration. Based on these results, iron deposition is now considered as a secondary alteration in dopaminergic neuronal death in PD.

### Oxidative stress and Parkinson’s disease

Oxidative damage is an important factor in the nigral neuronal death. Members of Prof. Mizuno’s lab, Dr. Yoritaka, and Dr. Shimura, found an increase in 4-hydroxynonenal-modified protein, (Yoritaka et al. 1996) 8-oxo-dGTPase, and 8-oxo-7,8-deoxyguanosine (Shimura-Miura et al. 1999), in the SN of the PD brain. These studies highlight the importance of oxidative stress-induced mitochondrial damage in the pathogenesis of nigral neuronal death in PD. Vitamin E (alpha-tocopherol) is a potent antioxidant in the cell membrane that can trap free radicals and prohibit lipid peroxidation. To investigate the effect of vitamin E deficiency on the development of PD, Dr. Ren generated an MPTP model of PD, using alpha-tocopherol transfer protein (TTP) knockout (TTP–/–) mice (Ren et al. 2006). MPTP treatment tended to decrease striatal dopamine levels; however, the effect was comparable and not significant for any specific genotype. We also examined the effect of oral vitamin E. Oral administration of vitamin E resulted in partial protection of striatal dopaminergic terminals against MPTP toxicity. Our results suggest that vitamin E does not play a major protective role against MPTP-induced nigrostriatal dopaminergic neurodegeneration in the brain. Similar to our results, clinical trials of tocopherol in PD have shown no therapeutic effects (Parkinson Study Group 1993). Oxidative stress cascades, other than that of vitamin E may have a greater impact on PD pathogenesis.

### Apoptosis, mitochondria, and Parkinson’s disease

We investigated cell death mechanism induced by MPP^+^, a metabolite of MPTP, in a dopaminergic neuron culture system(Nishi et al. 1994). We observed that degeneration occurred from the neurite terminal. Conversely, Youle et al. observed internucleolar DNA degradation when MPP^+^ was administered to a cerebellar granule neuron culture system (Dipasquale et al. 1991). As cerebellar granule cell cultures are not a suitable model for PD, we examined nuclear changes in dopaminergic neuron cultures by TdT-mediated dUTP Nick-End Labeling (TUNEL) assay and DNA electrophoresis and detected apoptosis characterized by alterations in nuclear morphology and DNA fragmentation (Mochizuki et al. 1994b). Owing to these unexpected results, we next used the TUNEL method to study the neurons in the SN of autopsied PD brains; the apoptosis process presented by nuclear staining was observed in eight out of 11 parkinsonian patients studied (Mochizuki et al. 1996). This suggests that PD results from inappropriate activation of cell death by apoptosis.

Although the link between mitochondrial dysfunction and apoptosis was initially unclear, new associations have been reported, as described below. Various signals mediating cell death are initiated by the release of cytochrome c due to mitochondrial damage. This pathway requires the apoptotic protease-activating factor-1 (Apaf-1), which is responsible for the recruitment of procaspase-9. In the presence of dATP and cytochrome c, a 1:1 complex is formed between Apaf-1 and procaspase-9, which mediates the activation of caspase-9 via oligomerization, followed by the activation of downstream caspases, leading to cell death. Using a PD model, we investigated whether suppression of this system would prevent neuronal cell death.

Starting with inhibition of the mitochondrial apoptotic cascade, under the guidance of Prof. Miura, we created a recombinant adeno-associated virus (rAAV) vector containing the caspase recruitment domain of Apaf-1, which was used to inhibit the formation of the functional Apaf-1-caspase-9 complex. rAAV-Apaf-1-DN was optimized to inhibit cell death via the Apaf-1/caspase-9 pathway. When rAAV-Apaf-1-DN was administered unilaterally to the SN of the MPTP model, only the administered side evaded cell death (Mochizuki et al. 2001). Therefore, we demonstrated that the primary mechanism of MPTP-induced dopaminergic neuronal cell death induced by MPTP is the mitochondrial apoptotic pathway.

The discovery of parkin was a major breakthrough in Prof. Mizuno’s laboratory (Kitada et al. 1998). Parkin functions primarily as an E3 ubiquitin ligase, catalyzing K48-bound polyubiquitination and proteasomal degradation of substrates (Shimura et al. 2001). Several studies have also shown that there is a functional interaction between parkin and PINK1, which is involved in mitochondrial quality control (Vives-Bauza et al. 2010; Matsuda et al. 2010). It is well known that parkin tags damage mitochondria with ubiquitin and activate autophagic degradation during mitophagy. This is also an important mechanism for cell death and Parkinson’s disease. Thus, overexpression of parkin may also be therapeutic in terms of mitochondrial regulation. In our experiments, overexpression of parkin protected dopaminergic neurons in a chronic MPTP model but did not affect the elimination of potentially harmful mitochondrial accumulation (Yasuda et al. 2011). Further studies are needed.

Granulocyte colony-stimulating factor (G-CSF) is a growth factor that acts as a neurotrophic factor. In fact, G-CSF receptors are expressed in neurons in the brain and spinal cord. The action of G-CSF on the central nervous system induces neurogenesis, enhances neural plasticity, and counteracts apoptosis. We examined whether G-CSF protected dopaminergic neurons against MPTP-induced cell death in a mouse model of PD. Our findings indicate that G-CSF provides neuroprotection against MPTP-induced cell death and that this effect is mediated by increased Bcl-2 expression and decreased Bax expression levels in C57BL/6 mice (Cao et al. 2006). Based on these results, 11 patients with atypical parkinsonism (4 patients with multiple system atrophy (MSA), 5 patients with progressive supranuclear palsy (PSP), 2 patients with corticobasal degeneration (CBD) were treated with G-CSF (5 mcg/kg daily for 6 days/month) for 3 months in Italy. CBC, CD34 + cells, routine biochemical tests, coagulation tests, UPDRS motor scores, and safety were evaluated. No serious adverse events were observed during or after G-CSF administration. Additionally, G-CSF can be safely administered to patients with atypical parkinsonism, and potentially meaningful clinical changes can be observed in some patients (Pezzoli et al. 2010).

### Neuroinflammation and Parkinson’s disease

McGeer et al. reported the activation of HLA-DR-positive microglia in the SN, as a pathological finding of neuronal death in PD (McGeer et al. 1988). This indicated that local inflammation is induced in the SN in PD. In PD, increased expression of interleukin (IL)-1β was already reported in striatum, CSF, and peripheral blood mononuclear cells; increased IL-1β expression has been used as a sensitive and specific marker of caspase-1 activation since caspase-1 is the major activator of pro-IL-1β. We succeeded in creating two models of cell death by modifying the method of MPTP administration, a chronic model which involves apoptosis and an acute model which provokes an inflammatory response. Caspase 11 KO mice, which have suppressed inflammatory pathways, are resistant to inflammatory PD-like pathologies that result in acute but not chronic MPTP administration (Furuya et al. 2004).

Why does local inflammation occur in patients with PD? Severe pneumonia can exacerbate PD symptoms even after complete recovery. Lipopolysaccharide (LPS), a component of the cell wall of gram-negative bacteria, induces inflammation and activates microglia and is also known to pass through the blood–brain barrier (BBB). We successfully created a model of unilateral PD, in which dopaminergic neurons in the SN died when very small doses of LPS were administered to the SN. Intranigral injection of LPS decreased the number of tyrosine hydroxylase-positive neurons and increased the number of microglial cells in the SN compared to that in the contralateral side. Expression of caspase-11 mRNA in the ventral midbrain and caspase-11-positive cells in the ipsilateral SN were detected in this mouse model. LPS injection failed to elicit these responses in caspase-11 knockout mice (Arai et al. 2004). This neuroinflammatory pathway may exacerbate the symptoms of PD, which are complicated by serious infectious diseases (Arai et al. 2006).

### α-Synuclein toxicity and Parkinson’s disease

α-Synuclein (αSyn) plays an important role in several types of PD. Indeed, αSyn is present in LBs, a pathognomonic feature of PD, and point mutations in the αSyn gene (PARK1) and triplication of the αSyn locus (PARK4) can be etiologic in rare cases of familial PD. At that time, transgenic mice for αSyn had already been developed; however, cell death was not so marked in this and hence, was not a good PD model. Our next step was to create a PD model related to αSyn. Using the rAAV vector system to introduce the human synuclein gene into the rat SN, we observed an approximately 50% loss of dopaminergic neurons 13 weeks after administration. During the slow progression of neurodegeneration, we identified several important features common to the pathogenesis of PD, including the phosphorylation of αSyn at Ser129 and activation of caspase-9(Yamada et al. 2004).

We examined the association between ubiquitin carboxy-terminal hydrolase L1 (UCH-L1) and cell death in a mouse model of PD-overexpressing αSyn. Our study showed that accumulated αSyn is neurotoxic to DA neurons and that such neuro-toxicity is enhanced by PARK5-associated UCH-L1 Ile93Met mutant, but not influenced by the loss of UCH-L1 wild-type protein in vivo. Next, we examined the association between parkin and cell death in a primate model of PD-overexpressing αSyn (Yasuda et al. 2009). In mice and primates, overexpression of αSyn induced neuronal loss, whereas co-expression of parkin alleviated αSyn toxicity. These results suggest that the inhibitory effect of parkin on αSyn, is exerted on neurons in mice and primates (Yamada et al. 2005; Yasuda et al. 2007).

### α-Synuclein function and Parkinson’s disease

In 2010, I moved to the Department of Neurology, Kitasato University School of Medicine, as professor and chairman. I established the Endowed Chair in Neuro Regenerative Medicine in 2011 and invited Prof. Mizuno as a project professor at Kitasato University.

Our interests still lie in the physiological function of αSyn. Pathological examination of patients with dementia with LBs has confirmed the presence of abnormal αSyn aggregates at presynaptic terminals; αSyn is abundantly localized at presynaptic terminals. αSyn regulates the soluble *N*-ethylmaleimide-sensitive factor attachment protein receptor (SNARE) complex. Importantly, postmortem examinations of αSyn transgenic mice and patients with PD demonstrated an abnormal distribution of SNARE proteins in presynaptic terminals. Next, using SNAP25 S187A/S187A mutant mice, Dr. Nakata from our group examined in detail, the effects of SNARE dysfunction on endogenous αSyn, in collaboration with Prof. Takahashi of the Department of Biochemistry, Kitasato University. In conclusion, this study shows that SNARE dysfunction leads to the accumulation of endogenous αSyn in corticostriatal nerve terminals; presynaptic accumulation of αSyn is considered an important early event in the pathogenesis of α-synucleinopathies (Nakata et al. 2012). Furthermore, SNAP25 is well known to be reduced in the striatum of MSA brains, suggesting that the discontinuous pattern of synaptic pathology normally observed in MSA may be related to presynaptic accumulation in corticospinal neurons (Yasuda et al. 2013).

Dr. Miyakawa from our department reported the neuropathological findings in an older patient with a homozygous deletion of parkin’s exons 2–4. An autopsy revealed a marked reduction in the number of melanized neurons in the SN and spinal cord loci. In patients with PARK2, LB formation with αSyn accumulation is usually absent. However, in this patient, the LB was found in the SN, locus coeruleus, dorsal motor nucleus of the vagus, basal ganglia of Meynert, amygdala nucleus, and sympathetic bundles of the cardiac muscle (Miyakawa et al. 2013). No accumulation of αSyn was detected in induced pluripotent stem cells (iPSC)-derived neurons from young Park2 patients. However, in iPSC-derived neurons from the reported patient, an accumulation of αSyn was observed (Imaizumi et al. 2012). Thus, the correlation between parkin deficiency and αSyn aggregation is not so straightforward.

Prof. Mizuno has a keen interest in the cell death mechanism in MSA, especially the correlation between αSyn and cell death-related proteins and the sequential changes of αSyn in the MSA brain. Hence, Dr. Hayakawa sequentially evaluated the immunohistochemical reactivity of αSyn, phosphorylated αSyn (pαSyn), dopamine and cAMP-regulated phosphoprotein 32 kDa (DARPP-32), calbindin-D 28 k, calpain cleaved carboxy-terminal 150 kDa spectrin fragment, and tyrosine hydroxylase in MSA autopsied brains. Immunohistochemical reactivity of αSyn, pαSyn, or both were elevated in all regions examined in MSA oligodendrocytes. Decreased immunostaining for DARPP-32 and calbindin-D 28 k was most evident in the posterior putamen, where neuronal loss was most prominent. It was also observed in the anterior putamen and caudate head, where neuronal loss was less prominent or absent. Calbindin immunostaining was also decreased in the dorsal tiers of the SN and cerebellar cortex. The reduced immunostaining for DARPP-32 and calbindin-D 28 k observed before neurodegeneration indicates that the loss of calbindin-D precedes the loss of neurons and that calcium toxicity and derangement of the protein phosphorylation state are relatively early events in the development of MSA (Hayakawa et al. 2013).

### α-Synuclein structure and Parkinson’s disease

Finally, I was appointed professor and chairman of the Department of Neurology at Osaka University, where my team focused on examining the structure of αSyn to determine exactly how the protein structure looked like in PD autopsied brains. In particular, it is important to find out if αSyn has an amyloid structure. At this point, the mode of protein propagation was still unknown, and LBs were not stained by Congo red staining, so it was thought that αSyn consists of no amyloid fibers. Since electron microscopy does not provide information on the secondary structure of the protein, it was not known whether LB has a *β*-sheet structure. To confirm that LB contains amyloid fibrils, the amount of *β*-sheet present would have to be confirmed. Fourier Transform Infrared Spectroscopy (FTIR) is a well-established structural analysis method that is sensitive to the secondary structure of a protein; it shows spectra derived from chemical bonds and thus provides detailed structural information that cannot be obtained by staining or EM. However, FTIR measurements of LB are difficult to perform. The greatest difficulty is that LBs are too small to be illuminated by an infrared beam, and their density is too low to produce a significant signal.

To overcome this problem, a strong and small infrared beam is required. We used synchrotron radiation from the synchrotron radiation facility at SPring-8 (Amagasaki, Hyogo Prefecture, Japan). Dr. Araki from our department presented the world’s first data on the secondary structure of LBs, using synchrotron radiation FTIR microspectroscopy. In addition, *β*-sheet mapping was performed to elucidate LB formation. Our results showed a shift in the infrared spectrum that indicates abundance of *β*-sheet-rich structure in LBs. Furthermore, 2D infrared mapping of LBs revealed that the content of the *β*-sheet structure is higher in the halo than in the core, and the core contains a large amount of proteins and lipids (Araki et al. 2015). More importantly, using these methods, we found structural differences between LBs in patients with PD and glial cell inclusions in patients with MSA (Araki et al. 2020). These structural differences may provide clues to the differences between the phenotypes of PD and MSA.

Isolated A*β* and αSyn proteins can aggregate when incubated in vitro for several days to form amyloid fibrils with a cross-*β* structure. However, the presence of cross-*β* sheet-rich aggregates in LBs has not been experimentally demonstrated so far. Dr. Araki also examined LBs in thin sections of autopsied brains of patients with PD using microbeam X-ray diffraction at SPring-8. Interestingly, he found that some of the LBs gave a diffraction pattern typical of a cross-*β* structure (Araki et al. 2019). This result confirmed that LBs in the brain of patients with PD contained amyloid fibrils with a cross-*β* structure and supported the validity of in vitro propagatin experiments using artificially formed amyloid fibrils of αSyn.

To detect the amyloid fibrils in PD, Prof. Goto, our collaborator, established a high-throughput ultrasonication-induced amyloid fibrillation assay (HANABI) to amplify and detect αSyn aggregates from cerebrospinal fluid (CSF), and Dr. Kakuda from our department investigated the correlation between seeding activity and clinical indicators. The CSF from patients with PD showed higher seeding activity than that from control patients. These findings showed that our HANABI assay can rapidly amplify misfolded αSyn and can be used to evaluate the seeding activity of CSF (Kakuda et al. 2019).

### α-Synuclein propagation and Parkinson’s disease

To identify the mechanism underlying αSyn propagation, we studied several models focusing on the intracellular and extracellular kinetics of αSyn.

**Fig. 1:**
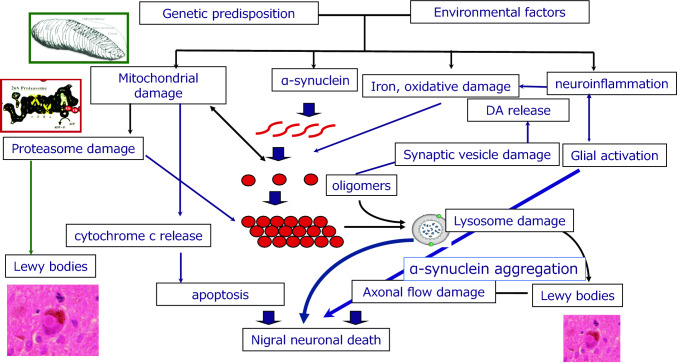
Model for pathogenesis of sporadic Parkinson’s disease. In Parkinson’s disease, mitochondrial dysfunction, iron, oxidative stress, neuroinflammation, and proteasome dysfunction could induce aggregation of α-synuclein, with subsequent formation of intermediate filaments of α-synuclein. Finally, Lewy bodies are formed although whether their formation is cytotoxic is debatable

A patient with αSyn G51D mutation in Japan exhibited rapid and severe clinical symptoms (Tokutake et al. 2014). To know the differences between sPD and PD with G51D mutation, Dr. Baba and his colleagues in our department, examined the mechanisms associated with severe neurotoxicity of αSyn G51D mutation using a murine model that was generated by G51D αSyn fibril injection into the brain. They found that G51D αSyn fibrils have higher *β*-sheet content than that of wild-type αSyn fibrils. The addition of G51D αSyn fibrils to mammalian cells overexpressing αSyn resulted in the formation of phosphorylated αSyn inclusions at a higher rate. Similarly, injection of G51D αSyn fibrils into the SN of mouse brain induced more widespread phosphorylated αSyn pathology. Notably, the mice injected with G51D αSyn fibrils exhibited progressive nigral neuronal loss accompanied with mitochondrial abnormalities and motor impairment. Their findings indicate that the structural difference of G51D αSyn fibrils play an important role in the rapid progression and severe neurotoxicity of G51D mutation-linked PD (Hayakawa et al. 2020). This model is also suitable for the evaluation of the propagation mechanisms of αSyn (Fig.1).

Next, we examined the extracellular transport pathways of αSyn. Genome-wide association studies have revealed that human leukocyte antigen (HLA) class II is a PD-associated gene; however, the mechanisms linking HLA class II and PD remain elusive. Dr. Ozono from our department, collaborating with Prof Arase, identified a novel function of HLA class II in the transport of intracellular αSyn to cell exterior. HLA class II molecules and αSyn formed complexes and moved to the cell surface at various degrees among HLA-DR alleles. Inhibition of complex formation via the peptide binding groove of HLA class II molecules and the N-terminal side of αSyn may provide a potential therapeutic target for PD with HLA- DRB5*01:01 risk alleles (Ozono et al. 2023).

We were also interested in studying the initiation of αSyn aggregation. Several studies have examined the association between the interaction of αSyn with lipids, particularly glucosylceramide, and the propensity of αSyn to aggregate. In our previous study, structural analysis of LBs in the PD brain revealed that lipids were abundantly distributed in the core of LBs, even in patients with idiopathic PD, indicating the involvement of some lipids in the initiation of αSyn aggregation (Araki et al. 2015). A subsequent study aimed to identify the lipid molecules and mechanisms involved in the physiological and pathological changes in αSyn in PD. Cell-based assays showed that upregulation of PIP_3_ in cells induces the formation of αSyn inclusions. In vitro protein-lipid overlay and aggregation assays further confirmed that PIP_3_ is a lipid molecule that directly interacts with αSyn monomers, initiates aggregation, and induces the formation of PD-like fibrils. In neurons, elevated cellular PIP_3_ can recruit endogenous αSyn into forming pathologic inclusions in the presynaptic regions. Taken together, PIP_3_ dysregulation promotes the pathological aggregation of αSyn and increases the risk of developing PD, and thus is a potent target for intervention in PD. These important studies were mainly conducted by Dr. Choong of our group (Choong et al. 2023).

Next, we focused on the study of intracellular αSyn aggregation to determine how endocytosed misfolded αSyn encounters normal αSyn molecules in the cytosol. Recent studies have shown that extracellular αSyn aggregates incorporated into the endosomal-lysosomal system can rupture the vesicular membrane of lysosomes. To investigate if lysosome rupture leads to the propagation of αSyn aggregation, Dr. Kakuda employed a cell-based model of αSyn aggregation propagation and showed that ruptured lysosomes represent a pathway for transmitting aggregation of exogenous αSyn aggregates and this process is prevented by lysophagy, i.e., selective autophagy of damaged lysosomes. αSyn aggregates are mainly accumulated in lysosomes, causing lysosome rupture and initially seeding endogenous αSyn aggregates around damaged lysosomes. Exogenous αSyn aggregates induce LC3 accumulation in lysosomes. This accumulation of LC3 was abrogated in cells lacking RB1CC1/FIP200, a key regulator of autophagy. Importantly, RB1CC1/FIP200-deficient cells treated with αSyn aggregates showed an increased number of ruptured lysosomes and enhanced propagation of αSyn aggregation. These results indicate that lysophagy prevents exogenous αSyn aggregates from escaping the endosomal-lysosomal system and transmitting aggregation to endogenous cytoplasmic αSyn via ruptured lysosomal vesicles. His findings suggested that the progression and severity of synucleinopathy are associated with damage to lysosomal membranes and impaired lysophagy (Kakuda et al. 2024).

### Future therapy for Parkinson’s disease

sPD is triggered by environmental and genetic factors, as well as aging. However, the exact triggers for this remain unknown. As mentioned above, mitochondrial dysfunction, oxidative stress and calcium homeostasis, cell death cascade, neuroinflammation, pathological glial activation, formation and amplification of intracellular α-Syn multimeric bodies and aggregates, protein homeostasis, lysosomal dysfunction, autophagy dysfunction, intercellular pathological α-Syn propagation, and other factors are involved in the pathogenesis of PD. Disease-modifying therapies target the molecular pathogenesis of sPD to inhibit or halt its progression, whereas symptom-improving therapies supplement the failing nervous system with dopamine replacement to improve symptoms. In the future, tight regulation of all these factors will lead to PD-modifying therapies; we present some of our data in this regard.

## Parkinson’s disease treatment by mitochondrial regulation

As mentioned previously, we initially focused on the regulation of the cell death cascade resulting from mitochondrial damage (Mochizuki et al. 2001). The regulation of cell death by Apaf-1-dominant negative showed significant improvement in the PD model, suggesting that it could be a promising therapeutic strategy for other neurodegenerative disorders.

Next, we focused on PCG-1α, a master regulator of mitochondrial biogenesis and function. However, it has been very challenging to protect mitochondrial biosynthesis by direct regulation of PGC-1α in neurons. We have identified necdin as a potent PGC-1α stabilizer that promotes mitochondrial biogenesis via PGC-1α in mammalian neurons in collaboration with Prof. Yoshikawa. Overexpression of necdin in the SN of adult mice protected against MPTP-induced degeneration of dopaminergic neurons. This data indicates that necdin promotes mitochondrial biogenesis through stabilization of endogenous PGC-1α and exerts neuroprotection against mitochondrial damage (Hasegawa et al. 2016).

It is well known that parkin tags damage mitochondria with ubiquitin and activate autophagic degradation during mitophagy. Therefore, parkin overexpression may also be therapeutic in terms of mitochondrial regulation. In our experiments, parkin overexpression conferred protection to dopaminergic neurons in a chronic MPTP model, but was not effective in eliminating the potentially harmful accumulation of mitochondria (Yasuda et al. 2011).

Recently, mitochondria have been shown to exist outside the cell, in free mitochondrial DNA, in functional or damaged mitochondria, and in extracellular vesicles, and may be involved in the pathogenesis of PD (Choong et al. 2021). There has also been a dramatic shift in the perception that the mitochondria are merely the power source of the cell. In addition to being essential to the cell, mitochondria promote cell repair when transplanted from healthy cells to damaged cells; hence, the therapeutic use of mitochondrial transplantation has been explored.

## Regulation of oxidative stress, neuroinflammation, and glial activation

G-CSF is expected to exert neuroprotective effects by inducing neuronal regeneration, inhibiting neuronal apoptosis, mobilizing hematopoietic stem cells, regulating pro- and anti-inflammatory cytokines, and activating angiogenesis. G-CSF also showed a therapeutic effect in the MPTP model (Cao et al. 2006); however, no noticeable changes were observed in the European Open Trial of MSA (Pezzoli et al. 2010).

GLP-1 receptor agonists are therapeutic agents for type 2 diabetes mellitus and have shown anti-inflammatory, neuroprotective, and inhibitory effects on glial activation, in animal models of PD (Yun et al. 2018). Exenatide showed significant improvement in the MDS-UPDRS part III in a phase II study, and a phase III study is underway. Dr. Kimura and his colleagues in our department are also planning to start a phase II investigator-initiated trial of oral semaglutide for PD, in the fall of 2023 and expect to observe results in conjunction with other trials.

## Regulation of α-Synuclein accumulation and propagation

As already noted, accumulating evidence indicates that pathological αSyn and αSyn aggregates play a central role in the pathogenesis of PD and that their intercellular transmission is associated with disease progression. Therefore, inhibition of αSyn aggregation or depolymerization of αSyn aggregates has been considered a promising therapeutic approach to PD. Dr. Hideshima of Dr. Ikenaka’s group in our department used a two-step screening method to identify candidate αSyn aggregation inhibitors. Taking advantage of the high-throughput nature of our method, we screened 1262 FDA-approved compounds and found tannic acid (TA) to be the most effective candidate as an αSyn aggregation inhibitor (Hideshima et al. 2022). Although TA does not cross the BBB, it may have therapeutic effects such as αSyn elimination from the intestinal tract; future studies are anticipated.

Among the existing αSyn-targeting therapies, antibody drugs against αSyn have shown great promise. The results of a phase II study on a high-profile antibody drug in 2022 have been reported. Both the SPARK study, which validated an anti-αSyn antibody, and the PASADENA study, which validated an anti-αSyn aggregate antibody, failed to meet their primary endpoints (Whone 2022). The main reason antibody therapy does not work is that αSyn has different structures depending on the disease. Ribozymes and antisense oligonucleotides (ASO) targeting αSyn mRNA remain the most promising therapies (Hayashita-Kinoh et al. 2006; Uehara et al. 2019). We are developing ASO-αSyn in collaboration with Prof. Obika of Osaka University’s Faculty of Pharmaceutical Sciences. Prof. Obika designed and synthesized an amido-bridged nucleic acid (AmNA)-modified ASO that targeted SNCA with improved stability and cellular uptake in vivo (Uehara et al. 2019). The current task is to raise the safety level and efficacy of ASO-αSyn for clinical research on humans.

Several important aspects should be considered for future clinical trials. The need for early treatment is best illustrated by the failure of recent clinical trials. Currently, pre-onset treatment is not ethically justified as standard treatment. Therefore, in order to enable early clinical intervention in the current situation, we have developed the sophisticated concept of recognizing “αSyn dysregulation disease (ASDD)” as “a pathological state in which αSyn protein aggregates abnormally accumulate before irreversible severe brain damage or symptom onset.” The concept of using the new term “ASDD” to describe the “pathological state of abnormal αSyn accumulation in the brain prior to irreversible severe brain damage or symptom expression” has led to the development of antibodies, vaccines, ASO, and small molecules to reduce αSyn protein levels and aggregates in at-risk prodromal patients to enable early clinical intervention (Mochizuki et al. 2018).

## Stem cell transplantation, autophagy regulators, and others

Recently, remarkable progress has been made in the development of stem cell-based therapies for PD. In Japan, in particular, the development of iPSC-based therapies has been progressing. In an open-label study in Japan, five patients received allogeneic iPSC-derived dopaminergic progenitor cells (from a stem cell bank with different HLA compatibilities with the host) to evaluate safety and efficacy (Barbuti et al. 2021).

Autophagy is a normal cellular mechanism for the removal of damaged organelles and aggregated proteins, and is involved in the pathophysiology of this disease. Our collaborator, Prof. Yoshimori showed that the expression of Rubicon, a negative regulator of autophagy, is increased at the transcript and/or protein levels in aged worms, flies, and mouse tissues, and that an age-dependent increase in Rubicon impairs autophagy over time, resulting in a reduced healthy lifespan of the animals. In Rubicon-knockout mice, αSyn accumulation in the brain was reduced (Nakamura et al. 2019). Collectively, these results suggest that Rubicon suppression is a candidate for PD therapy.

Disease-modifying drugs such as gene and antibody therapies, although very useful, are expensive and cannot be administered to many patients. In the future, people will be able to find drugs with high efficacy from supplements, herbal remedies, or drugs already on the market to prevent PD. As mentioned earlier, the TA is one such example (Hideshima et al. 2022). Dr. Jiang S, in collaboration with Prof. Hagihara, reported that Go-sha-jinki-Gan, a traditional Japanese herbal medicine with an existing role, attenuates inflammation of the central nervous system by suppressing glial cell activation, and thus, has the potential to be a therapeutic agent for PD (Jiang et al. 2021).

In summary, although the exact pathogenesis of PD remains unclear, the discovery of mitochondrial damage in PD, pioneered by Prof. Mizuno, has notably contributed to our understanding of the pathogenesis of PD. Furthermore, elucidation of the structural biology of αSyn has deepened our understanding of PD. These insights have not only led to the development of novel therapies targeting αSyn but may also pave the way for improved therapeutic intervention and better outcomes for patients with PD.

## Data Availability

Not applicable.

## Abbreviations

PD: Parkinson’s disease
MPTP: 1-Methyl-4-phenyl-l,2,3,6-tetrahydropyridine
αSyn: α-Synuclein
SN: Substantia nigra
MSA: Multiple system atrophy
PSP: Progressive supranuclear palsy
CBD: Corticobasal degeneration
